# The relationship between time to a high COVID-19 response level and timing of peak daily incidence: an analysis of governments’ Stringency Index from 148 countries

**DOI:** 10.1186/s40249-021-00880-x

**Published:** 2021-07-05

**Authors:** Yan Ma, Shiva Raj Mishra, Xi-Kun Han, Dong-Shan Zhu

**Affiliations:** 1 Institute of Basic Research in Clinical Medicine, China Academy of Chinese Medical Sciences, Beijing, China; 2 World Heart Federation, Rue de Malatrex 32, 1201 Geneva, Switzerland; 3 Nepal Development Society, Chitwan, Bharatpur-10, Bagmati Province Nepal; 4 Statistical Genetics, QIMR Berghofer Medical Research Institute, Brisbane, Australia; 5 School of Medicine, University of Queensland, Brisbane, Australia; 6 Centre for Health Management and Policy Research, School of Public Health, Cheeloo College of Medicine, Shandong University, 44 Wenhuaxi Road, Jinan, 250012 Shandong China; 7 NHC Key Lab of Health Economics and Policy Research (Shandong University), Jinan, 250012 China

**Keywords:** COVID-19, Response, Stringency Index, Multivariable linear regression models

## Abstract

**Background:**

The transmission dynamics and severity of coronavirus disease 2019 (COVID-19) pandemic is different across countries or regions. Differences in governments’ policy responses may explain some of these differences. We aimed to compare worldwide government responses to the spread of COVID-19, to examine the relationship between response level, response timing and the epidemic trajectory.

**Methods:**

Free publicly-accessible data collected by the Coronavirus Government Response Tracker (OxCGRT) were used. Nine sub-indicators reflecting government response from 148 countries were collected systematically from January 1 to May 1, 2020. The sub-indicators were scored and were aggregated into a common Stringency Index (SI, a value between 0 and 100) that reflects the overall stringency of the government’s response in a daily basis. Group-based trajectory modelling method was used to identify trajectories of SI. Multivariable linear regression models were used to analyse the association between time to reach a high-level SI and time to the peak number of daily new cases.

**Results:**

Our results identified four trajectories of response in the spread of COVID-19 based on when the response was initiated: before January 13, from January 13 to February 12, from February 12 to March 11, and the last stage—from March 11 (the day WHO declared a pandemic of COVID-19) on going. Governments’ responses were upgraded with further spread of COVID-19 but varied substantially across countries. After the adjustment of SI level, geographical region and initiation stages, each day earlier to a high SI level (SI > 80) from the start of response was associated with 0.44 (standard error: 0.08, *P* < 0.001, R^2^ = 0.65) days earlier to the peak number of daily new case. Also, each day earlier to a high SI level from the date of first reported case was associated with 0.65 (standard error: 0.08, *P* < 0.001, R^2^ = 0.42) days earlier to the peak number of daily new case.

**Conclusions:**

Early start of a high-level response to COVID-19 is associated with early arrival of the peak number of daily new cases. This may help to reduce the delays in flattening the epidemic curve to the low spread level.

**Graphic abstract:**

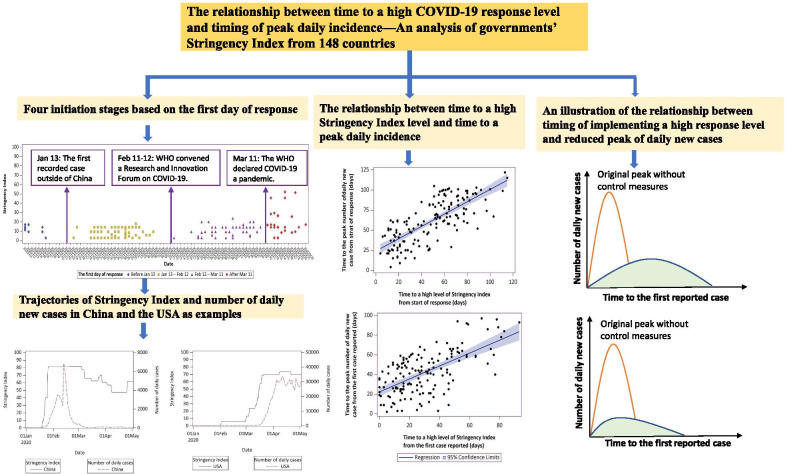

**Supplementary Information:**

The online version contains supplementary material available at 10.1186/s40249-021-00880-x.

## Background

The World Health Organization (WHO) declared COVID-19 a pandemic on March 11, 2020 [1]. Although several vaccines have been marketed and used, non-pharmaceutical interventions, such as social distancing, face masks, hygiene measures will be still key during the delivery of such vaccination programs [2, 3]. Social-control measures, combining epidemiological tactics to protect susceptible population are essential to curb the spread of COVID-19 [4–6].

The pandemic can be viewed as a series of distinct local epidemics in different spread phases. Countries experienced different sufferings during the spread. There are many reasons why the severity of the pandemic has varied in different countries or regions. In addition to the differences in capacity of countries to adapt their health system to the COVID-19 epidemic [7], differences in governmental policy responses may explain some of the differences. Governments around the globe have been taking a wide range of social control measures in response to the COVID-19 outbreak, such as school closure, and stay-at-home, etc. However, it’s hard to compare the measures implemented in different countries directly. The same measure might be implemented in different countries with different degree and intensity. And we lack a comprehensive index to reflect the overall response level of a country with implementation of multiple sub-measures.

Using publicly available data from the Coronavirus Government Response Tracker (OxCGRT), published by the Blavatnik School of Government at the University of Oxford [8, 9], we aim to track and compare worldwide government responses to the spread of COVID-19, to examine the relationship between a published response level index and the epidemic trajectories in 148 countries.

## Methods

### Data used

We used free publicly-accessible data collected by the Coronavirus Government Response Tracker (OxCGRT, https://www.bsg.ox.ac.uk/research/research-projects/covid-19-government-response-tracker). The OxCGRT systematically collects information from 148 countries on several policy responses that governments have taken, scores stringency of such measures, and aggregates them into a common Stringency Index (SI) in a daily basis [10, 11]. In the OxCGRT, the SI was calculated by using nine scaled indicators, including eight containment and closure policy indicators (school closing, workplace closing, cancel public events, restrictions on gatherings, close public transport, stay at home requirements, restrictions on internal movement, and international travel controls) and one indicators of public information campaigns. The definition of each indicator can be found elsewhere with a codebook (https://github.com/OxCGRT/covid-policy-tracker/blob/master/documentation/codebook.md). In brief, each indicator was transformed into a value between 0 and 100 according to the degree of response (e.g., cancelling a public event either received 0: if no measures was taken, 1: recommend cancelling, and 2: required cancelling). The value of the SI on any given day is the average value of these nine indicators. Thus, the index reports a number between 0 and 100 that reflects the overall stringency of the government’s response. Higher index indicates higher overall response level. A detailed description of the calculation of SI can be found in a file (https://www.bsg.ox.ac.uk/sites/default/files/Calculation%20and%20presentation%20of%20the%20Stringency%20Index.pdf). It also includes statistics on the number of reported COVID-19 cases in each country from the European Centre for Disease Prevention and Control (ECDC), and the Center for Systems Science and Engineering (CSSE) data repository at Johns Hopkins University. Our analyses used data from 148 countries and regions within the time span of 121 days from January 1, 2020 (the earliest date of OxCGRT record) to May 1, 2020.

### Dealing with missing data

In collecting the data of sub-indicators for calculating SI, the data for a certain indicator (e.g., stay at home) in some countries’ some days might be missing. This missing information cannot be interpreted as null or no measures adopted. This kind of incomplete or missing data can lead to reporting inaccuracies in calculated SI (usually a sudden dip). In our analysis, considering the governments’ implemented measures usually remain stable for some time, it was not possible to have a sudden dip or only last a couple of days (e.g., less than three days). Therefore, a sudden reduction in SI level or that lasting only for couple of days, was very likely due to missing data rather than a true reduction in stringency. We imputed the missing values or corrected these sudden dip SI by using moving average method. A moving average is a calculation used to analyze data points by creating a series of averages of different subsets of the full data set. By calculating the moving average, the impacts of random, short-term fluctuations on the SI level over a specified time-frame are mitigated.

### Statistical analysis

**Fig. 1:**
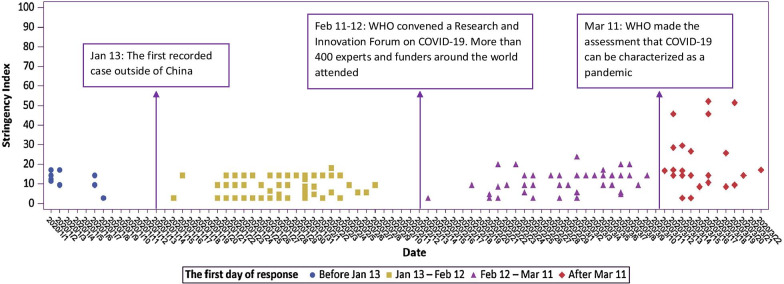
Four initiation stages based on the first day of response

We first plotted the date when response was first initiated (defined as SI > 0) and the level of initiated response from January 1, 2020 to May 1, 2020 in Fig. 1 to examine different stages of initiations.

Based on the dates collected, four time intervals were calculated: interval (1) was the days from the start of response (i.e., the first day of SI > 0) to the date of reaching a high level SI (defined as SI > 80); interval (2) was the days from the start of response to the date of the peak number of daily new case; interval (3) was the days from the first reported COVID-19 case to the date of reaching a high level of SI; and interval (4) was the days from the first reported COVID-19 case to the date of peak number of daily new cases. We fitted a linear regression model between interval (1) and (2), and between interval (3) and (4). Multivariable linear regression models were used to analyze the association between time to reach a high-level SI and time to the peak number of daily new cases, either from the start of response or from the date of first reported case. All models were adjusted for geographic regions of the world (including 19 regions of North America, South America, Caribbean, Central America, Central Asia, East Asia, Southeast Asia, Southern Asia, Western Asia, Eastern Europe, Western Europe, Southern Europe, Northern Europe, Oceania, Eastern Africa, Middle Africa, Northern Africa, Southern Africa, Western Africa), SI level and initiation stage. The model equation was as follows: Y = b0 + b1χ1 + b2χ2 + b3χ3 + b4χ4 + e. (Y: time to the peak number of daily new cases; χ1: time to reach a high-level SI; χ2: geographic regions; χ3: the highest SI level; χ4: initiation stage; b0: the constant; b1–b4: partial regression coefficient; e: random error). As linear regression models need a satisfaction of residual normality and no multicollinearity, we used Kolmogorov–Smirnov (K–S) method to test the normality of residual error, and the *P* value was 0.968. Thus, the residual error was approximately normally distributed. Multicollinearity was tested by using correlation matrix of independent variables, and no significant correlations were found among them (Additional file 1: Table 1). We also performed a sensitivity analysis redefining the start of a response with the first day of SI > 10.

We used group-based trajectory modelling (GBTM) method to examine daily trajectory of SI [12]. GBTM is designed to identify clusters of individuals (i.e. countries herein) following similar developmental trajectories of a single indicator of interest, i.e., SI in this study. We first specified one group to see the whole trajectory globally, then four groups were specified based on the imitation stage of countries. We also plotted the SI trajectory and number of daily new cases trajectory in 12 countries to observe the pattern of SI trajectory and their relationship with peak number of daily new cases. Last, we plotted the SI trajectory and number of daily new cases trajectory for all 19 geographic regions in the world.

SAS 9.4 (SAS Institute Inc., Cary, NC, USA) was used. Trajectory analysis was performed using the PROC TRAJ macro [13].

## Results

### Stages when a country initiated a response

By plotting distribution of first day of response (i.e., SI > 0) among 148 countries from January 1, 2020 to May 1, 2020, four initiation stages were observed (Fig. 1). The first stage is before January 13. Six countries or regions mainly from Asia, such as Singapore and Japan started a response during this stage. The second stage begins from January 13 (this is also the day when the first case outside of China was reported in Thailand) to February 12. Most of countries (74 countries from the 148 countries) started a response at stage 2, involving countries from all regions of the world. The third stage is from February 12 (when WHO convened a Research and Innovation Forum on COVID-19 and more than 400 experts and funders from around the world attended) to March 11, and there are 45 of 148 countries started a response at this stage. The last stage is from March 11 (the day WHO declared that COVID-19 as a pandemic) on going. There are 23 countries initiated a response at this stage, mainly from Eastern Africa and Western Africa (Additional file 1: Table 2). All governments of the 148 countries included had initiated a response by 22 March.

**Fig. 2:**
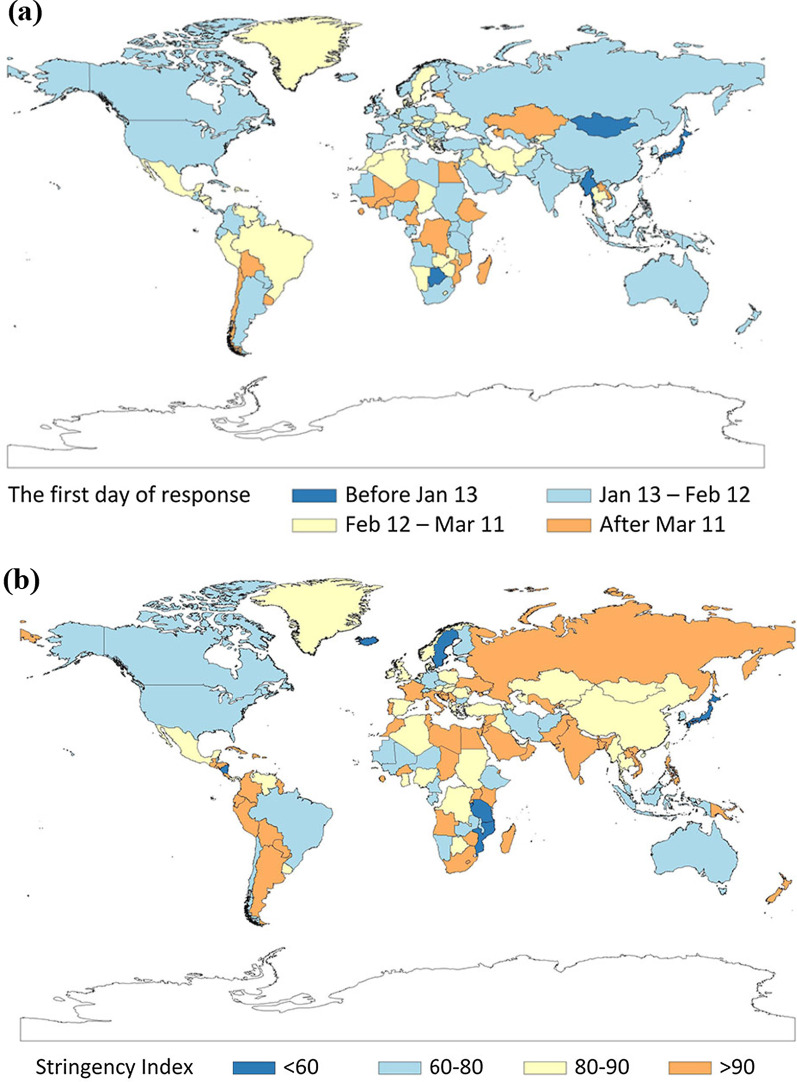
The timing of starting a response and level of response. **a** The first day of response to COVID-19 spread by country; **b** The highest level of Stringency Index by country

The timing of initiating a response by country was illustrated in Fig. 2a.

### The relationship between time to reach a high SI level and time to the peak daily number case

The mean initiation level of SI of countries at stage one to four were 12.4, 8.9, 11.6, and 20.4 respectively. The mean highest level of SI of countries at stage one to four were 74.6, 87.3, 84.2 and 83.7 respectively. The highest level of SI by countries were shown in Fig. 2b.

The average days from the start of response to the date of reaching a high SI level were 43.6 (standard deviation: 25.6) days, with median (Q1, Q3) of 47.0 (18.0, 61.0) days (Table 1). The average days from the start of response to the date of peak number of daily new cases were 61.5 (27.0) days, with median of 61.0 (38.0, 84.0) days. The average days from the first reported case to the date of reaching a high SI level was 26.0 (20.4) days, with median of 20.0 (12.0, 33.0) days. The average days from the date of first case reported to the date of peak number of daily new case was 41.9 (21.8) days, with median of 41.5 (25.0, 57.0) days (Table 1).

**Table 1:** Average days to a high Stringency Index (SI), peak number of daily new cases from starting a response, or from first reported case

Initiation stage	Days from first case reported to the date of reaching a high SI level		Days from first case reported to the date of peak number of daily new case		Days from starting a response to the date of reaching a high SI level		Days from starting a response to the date of peak number of daily new cases	
	Mean (SD)	Median (Q1, Q3)	Mean (SD)	Median (Q1, Q3)	Mean (SD)	Median (Q1, Q3)	Mean (SD)	Median (Q1, Q3)
Earlier than January 13, 2020	48.1 (30.2)	48.5 (25.0, 76.5)	48.2 (32.8)	58.0 (21.0, 66.0)	84.6 (22.1)	92.0 (77.0, 100)	92.0 (15.1)	96.0 (81.0, 100)
Between January 13 and February 12, 2020	29.9 (20.3)	25.0 (15.0, 48.0)	47.7 (21.0)	48.0 (34.0, 59.0)	58.0 (16.3)	57.5 (51.0, 65.0)	77.9 (19.2)	81.0 (66.0, 94.0)
Between February 13 and March 11, 2020	22.0 (17.3)	17.0 (13.0, 27.0)	37.4 (18.9)	36.0 (23.5, 49.0)	26.7 (14.4)	22.5 (16.5, 35.5)	44.5 (14.9)	45.0 (31.0, 58.5)
After March 11, 2020	13.2 (10.4)	9.5 (6.0, 16.0)	29.1 (17.9)	28.0 (17.0, 35.0)	13.2 (7.1)	13.0 (8.0, 16.0)	29.7 (16.4)	25.0 (16.0, 43.0)
Overall	26.0 (20.4)	20.0 (12.0, 33.0)	41.9 (21.8)	41.5 (25.0, 57.0)	43.6 (25.6)	47.0 (18.0, 61.0)	61.6 (27.0)	61.5 (38.0, 84.0)

*SD* standard deviation, *Q1* first quartile, *Q3* third quartile

**Fig. 3:**
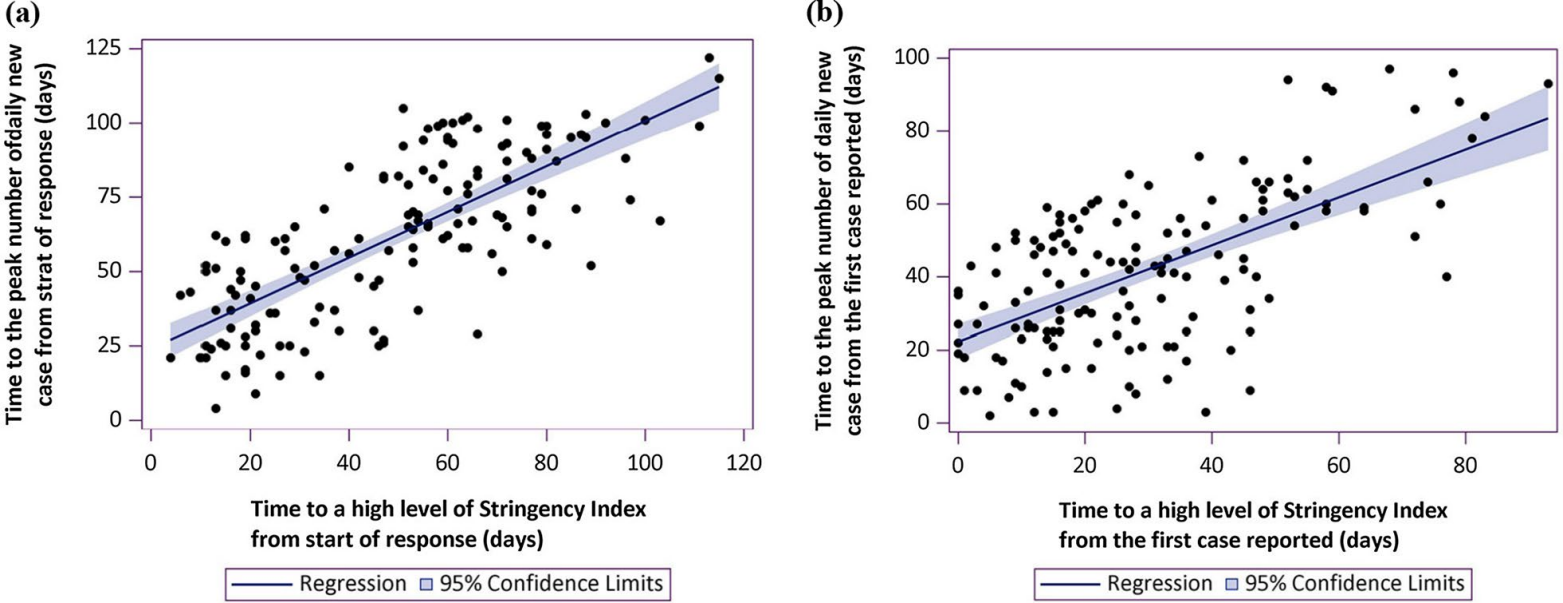
The relationship between time to a high Stringency Index level and time to a peak daily incidence. **a** When interval was calculated from the date of starting a response (R^2^ = 0.65, *P* < 0.001); **b** When interval was calculated from the date of first reported case (R^2^ = 0.42, *P* < 0.001)

Multivariable linear regression model showed that each day earlier to a high SI level (SI > 80) from the start of response was associated with 0.44 (standard error: 0.08, *P* < 0.001, R^2^ = 0.65) days earlier to the peak number of daily new case (Fig. 3a), after the adjustment of SI level, region and initiation stage. Also, each day earlier to a high SI level from the date of first reported case was associated with 0.65 (standard error: 0.08, *P* < 0.001, R^2^ = 0.42) days earlier to the peak number of daily new case (Fig. 3b). When a start of response was redefined as SI > 10, the results remained unchanged (Additional file 1: Table 3).

### The SI trajectory pattern and timing of peak daily incidence

**Fig. 4:**
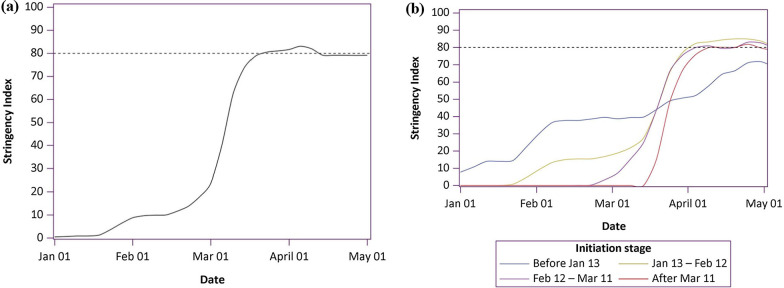
The trajectory of Stringency Index level. **a** The global level; **b** Based on initiation stages of countries

Trajectory analysis incorporating all 19 geographic regions showed three turning points consistent with the four stages we observed in Fig. 1 (Fig. 4a). Different trajectories of SI were identified in countries of different initiation stage (Fig. 4b). Countries in the 1st stage started a response early and keep a moderate SI level for a long period (e.g., Singapore and Japan). Countries that initiated at the 4th stage started quickly and upgraded to a high response level in a very short time (e.g. Egypt and Ethiopia). Countries who started at response at the 2nd and 3rd stage upgraded the SI level in a stepwise manner.

**Fig. 5:**
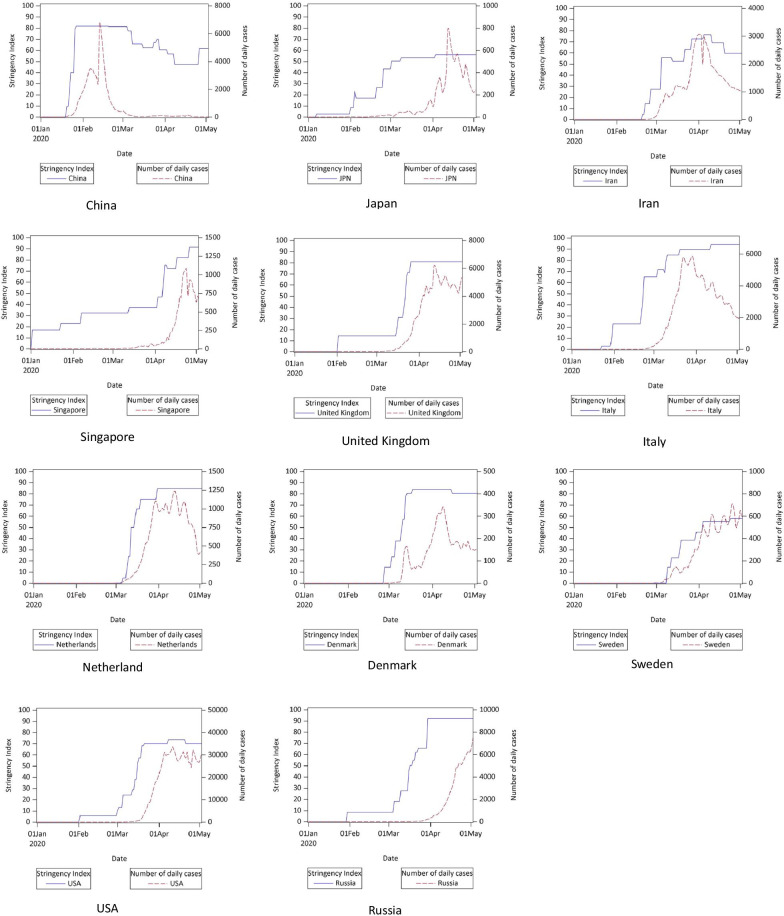
Trajectories of Stringency Index and number of daily new cases in 11 selected countries. An exponentially weighted moving average method with parameter 0.3 was used to smooth time series of Stringency Index and number of daily new cases, and a base-10 log scale was used for the Y axis of number of daily new cases

The specific trajectory of SI and number of daily new cases in 11 selected countries were shown in Fig. 5. Japan and Singapore initiated the response early and kept a low (SI < 50) to moderate SI for longer period, the time to the reduced peak daily new case was long (96 and 115 days respectively). China started a response and quickly raised to a high response level. Compared to Italy and the United Kingdom (UK) which reached a high-level SI late, China had an early appear of reduced peak number of daily new cases (24 days). Although the Netherlands and Denmark started a response in March, these countries were successful to upgrade to a high level in a short time. Unlike Iran, the Netherlands and Denmark took shorter time to reach reduced peak daily case (36 and 41 days respectively). Russia, the United States of America (USA) and Sweden took a long time to upgrade their response level. For the USA and Sweden with a highest SI level less than 80, daily case reached a high plateau.

The specific trajectory of SI and number of daily new cases in 19 geographical sub-regions of world were shown in Additional file 1: Fig. 1(a–d). In Asia, the spread of COVID-19 in East Asia was in a lower epidemic level than that in other Asian areas. This may benefit from quickly upgrading the response level to relatively high level. The spread in Oceania was controlled in a short time, which may be attributed to a quick implementation of a high response level (SI > 80). The spread in South America, Central America, Southern Asia, and most areas of Africa are still on a rise.

## Discussion

**Fig. 6:**
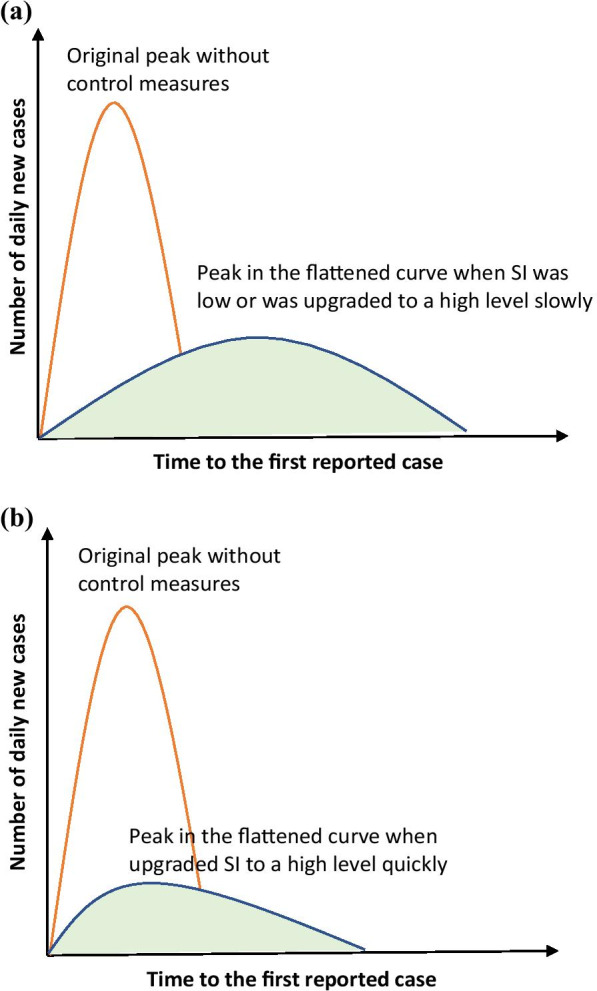
An illustration of the relationship between timing of implementing a high response level and reduced peak of daily new cases. **a** When stringency index (SI) was low or was upgraded to a high level slowly; **b** When upgraded SI to a high level quickly

Our results identified four trajectories of response in the spread of COVID-19 based on when the first response was initiated. Overall, governments’ responses were upgraded rapidly with further spread of COVID-19. However, these responses varied substantially across countries and regions. Shorter time interval to reach a high response level was associated with earlier arrival of a reduced peak of daily new case (as illustrated in Fig. 6).

Different initiation stages and trajectory of SI reflects the gradual shifts in the spread of COVID-19 both in space and time [14], also reflects whether a government response timely and vigilantly. For example, social-control measures that worked for COVID-19 in areas such as Singapore, Japan and China came later in Europe and the USA.

The countries who started a response at the first stage initiated early, and kept a relatively low or moderate response level (usually with a SI < 60) for a long time, such as Japan and Singapore. The countries who started a response at the second stage have a wide difference in the date of initiation and have a mixed initiation pattern. For instance, China started a response and upgraded to a high response level in a short time interval (less than 2 weeks). Other countries upgraded to a high-level response in a stepwise fashion with mixed scale. Compared to the countries who initiated a response at the second stage, countries which initiated a response at the third stage had relatively shorter time span of initiation, and the interval between initiation and upgrading to a high level was also shorter than that of countries at the second stage. The countries at the fourth stage (after declaration of pandemic by WHO) started the response in a very short time span and had the shortest interval from initiation to a high response level.

Except a few countries from Asia which adopted measures earlier and maintained a moderate response throughout (e.g., Singapore and Japan), the majority of other countries who started a response at the second, third and fourth stage, upgraded their response to a high level only after WHO declared COVID-19 as a pandemic. This indicates announcement of COVID-19 pandemic has triggered countries to act more aggressively against COVID-19.

Four patterns of relationships between trajectory of SI and daily case number were identified in our analyses. First, some Asian countries and regions (e.g., Singapore, and Japan) started response early (at the first stage) and kept a low or moderate SI level for longer period. The early initiation of a response helped these countries to flatten the original peak of epidemic curve [15, 16]. Nevertheless, the low or moderate response level make the peak number of daily new cases (after measures adopted) come later. Although a low to moderate response level can fight against the virus’ spread without major disruption to daily living [15], it bears some risk. In some countries such as Singapore, even a small rebound of cases can seriously jeopardize the existing efforts in curbing disease transmission, and need a serious concerted public response [17].

Second, some countries initiated and upgraded the SI to a high level in a short interval, and kept it for a while, such as China, Denmark and the Netherlands. The reduced peak daily incidence in these countries came earlier than countries where the first pattern was found. This implies a quick and aggressive social control measures not only can flatten the peak of epidemic curve (as shown in previous literature) [18–20], it also make the turning point of the epidemic curve come earlier (as illustrated in Fig. 6). Third, unlike countries in the second pattern, some countries initiated like Italy and Russia have taken a long time before reaching a high response level. The reduced peak of daily incidence was relatively high and only achieved at a later stage. Fourth, some countries started a response only at a later stage, and maintained a relatively low SI level (without achieving a high response level), such as Sweden and USA. In these countries, even the peak daily incidence in epidemic curve under intervention was high (or stay on a plateau for a period) and came later.

Social-control measures, medications and a vaccine are key weapons against the pandemic [21, 22]. Before herd immunity was formed in population by largescale vaccination, social-control measures, combing classical epidemiological tactics such as isolating the sick, quarantining their contacts are still highly relevant in the fight against COVID-19 [23]. Studies have reported that non-pharmaceutical interventions such as social distancing have substantially flattened the original peak (when no social measure was adopted) and area under the epidemic curve [16, 20], thus reducing the pressure on the health system from high daily incidence of COVID-19 cases. This might have also reduced the burden on health care workers who are currently working beyond the point of exhaustion.

The interval from the first reported case to the time of high response level (SI > 80) reflects the agility of a government’s response—depicting how quickly a government adopt comprehensive and aggressive measures, following the recommendation by the WHO [24]. Upgrading the response level to a high level in a short time (usually with SI greater than 80), can help to achieve a reduced peak of daily new case sooner than later. This also indicates an earlier arrival of turning point in the epidemic curve that can potentially prevent collateral damage and support health system recovery.

Different from previous studies which have focused on the effectiveness of non-pharmaceutical interventions on preventing COVID-19, we quantified the relationship between the speed of reaching a high response level and the timing of the peak of epidemic curve. This implies that both timing and intensity of the governments’ response affect the pandemic. Sooner to upgrade to a high SI level might be the optimal option to curb COVID-19 pandemic.

There are several limitations for the data we used. First, although we have dealt missing date with imputation method, it might still be inadequate to capture “real” data. For example, in China, the sub-indicator of staying at home requirement was missing between February 3 and April 7, 2020. We imputed the degree of this indicator by searching the implement status of this indicator during this period in China. Also, being a big country with many provinces or states, one parameter in the calculation of SI for indicating whether a measure was adopted in the whole country level (general) or in a certain area level (targeted) might cause some bias. Provinces could differ in implementing or canceling the same measure based on their COVID-19 epidemic situation. Further, same requirement may be carried out differently at personal level based on acceptability of the measure in different countries. Last, the capacity of case detection, the intensity of PCR test to actively find people who were infected, and the accuracy of case report varied from country to country. These might have caused confounding bias to the parametric estimates of regression.

## Conclusions

In conclusion, early start of a high-level response is associated with early arrival of the peak number of daily new cases (usually viewed as a turning point in the epidemic curve). This may help to reduce the delays in flattening the epidemic curve to the low spread level. Before a large-scale vaccination plan can be adopted, especially to low- and middle-income countries with insufficient vaccine stocks, non-pharmaceutical interventions are still key weapons to curb the epidemic. Upgrading governments' response to a high level timely not only can flatten the epidemic curve, but buy time for further stockpiling and vaccination.

## Supplementary Information

**Additional file 1: Table 1.** Correlation coefficient matrix of independent variables, r (p). **Table 2.** Stringency Index levels of countries based on different initiation stages. **Table 3.** The relationship between time to a high Stringency Index (SI) level and time to a peak daily incidence, when a start of response was redefined as SI > 10*. **Figure. 1A **Trajectories of Stringency Index and number of daily new cases in Asia. **B **Trajectories of Stringency Index and number of daily new cases in Europe. **C **Trajectories of Stringency Index and number of daily new cases in America, Oceania and Caribbean. **D **Trajectories of Stringency Index and number of daily new cases in Africa.

## Data Availability

Not applicable.
