# Supplementary material for: The relationship between time to a high COVID-19 response level and timing of peak daily incidence: an analysis of governments’ Stringency Index from 148 countries
Source: Infect Dis Poverty. 2021 Jul 5;10:96. doi: 10.1186/s40249-021-00880-x (PMC8256203; doi:10.1186/s40249-021-00880-x)
Supplement: Supplementary file 1 — Additional file 1: Table 1. Correlation coefficient matrix of independent variables, r (p). Table 2. Stringency Index levels of countries based on different initiation stages. Table 3. The relationship between time to a high Stringency Index (SI) level and time to a peak daily incidence, when a start of response was redefined as SI > 10*. Figure. 1A Trajectories of Stringency Index and number of daily new cases in Asia. B Trajectories of Stringency Index and number of daily new cases in Europe. C Trajectories of Stringency Index and number of daily new cases in America, Oceania and Caribbean. D Trajectories of Stringency Index and number of daily new cases in Africa. [file 40249_2021_880_MOESM1_ESM.docx]

| **Additional Table 1. Correlation coefficient matrix of independent variables, r (p)** | | | | |
| --- | --- | --- | --- | --- |
|  | Time to reach a high-level SI | Initiation stage | The highest SI level | Geographic regions |
| Time to reach a high-level SI | 1 | -0.58147(0.09) | 0.04023 (0.625) | -0.03145 (0.7024) |
| Initiation stage | | 1 | -0.06258 (0.4468) | -0.00731 (0.9293) |
| The highest SI level | | | 1 | 0.12023 (0.1428) |
| Geographic regions | | |  | 1 |
| SI, stringency index | | | | |

| **Additional Table 2. Stringency Index levels of countries based on different initiation stages** | | | | |
| --- | --- | --- | --- | --- |
| Country | Region | Stages of initiation | Stringency Index level at initiation | The highest Stringency Index level |
| Japan | East Asia | Before Jan 13 | 2.86 | 56.19 |
| Mongolia | East Asia | Before Jan 13 | 14.29 | 81.19 |
| Brunei | Southeast Asia | Before Jan 13 | 14.29 | 92.38 |
| Myanmar | Southeast Asia | Before Jan 13 | 9.52 | 83.1 |
| Singapore | Southeast Asia | Before Jan 13 | 17.14 | 91.43 |
| Botswana | Southern Africa | Before Jan 13 | 12.38 | 87.62 |
| Barbados | Caribbean | From Jan 13 to Feb 12 | 14.29 | 86.67 |
| Cuba | Caribbean | From Jan 13 to Feb 12 | 14.29 | 97.14 |
| Jamaica | Caribbean | From Jan 13 to Feb 12 | 8.57 | 75.95 |
| Trinidad and Tobago | Caribbean | From Jan 13 to Feb 12 | 5.71 | 92.38 |
| Costa Rica | Central America | From Jan 13 to Feb 12 | 14.29 | 97.14 |
| El Salvador | Central America | From Jan 13 to Feb 12 | 14.29 | 92.38 |
| Guatemala | Central America | From Jan 13 to Feb 12 | 2.86 | 97.14 |
| Panama | Central America | From Jan 13 to Feb 12 | 9.52 | 82.86 |
| Uzbekistan | Central Asia | From Jan 13 to Feb 12 | 14.29 | 92.38 |
| China | East Asia | From Jan 13 to Feb 12 | 9.52 | 81.72 |
| South Korea | East Asia | From Jan 13 to Feb 12 | 4.76 | 80.00 |
| Burundi | Eastern Africa | From Jan 13 to Feb 12 | 14.29 | 22.86 |
| Kenya | Eastern Africa | From Jan 13 to Feb 12 | 2.86 | 97.14 |
| Rwanda | Eastern Africa | From Jan 13 to Feb 12 | 14.29 | 97.14 |
| Seychelles | Eastern Africa | From Jan 13 to Feb 12 | 14.29 | 94.29 |
| Tanzania | Eastern Africa | From Jan 13 to Feb 12 | 2.86 | 51.43 |
| Uganda | Eastern Africa | From Jan 13 to Feb 12 | 2.86 | 94.29 |
| Bulgaria | Eastern Europe | From Jan 13 to Feb 12 | 2.86 | 72.86 |
| Poland | Eastern Europe | From Jan 13 to Feb 12 | 9.52 | 88.81 |
| Romania | Eastern Europe | From Jan 13 to Feb 12 | 2.86 | 85.24 |
| Russia | Eastern Europe | From Jan 13 to Feb 12 | 8.57 | 92.38 |
| Slovak Republic | Eastern Europe | From Jan 13 to Feb 12 | 4.76 | 88.81 |
| Angola | Middle Africa | From Jan 13 to Feb 12 | 5.71 | 92.38 |
| Gabon | Middle Africa | From Jan 13 to Feb 12 | 9.52 | 79.29 |
| Bermuda | North America | From Jan 13 to Feb 12 | 14.29 | 93.57 |
| Canada | North America | From Jan 13 to Feb 12 | 2.86 | 80.00 |
| United States | North America | From Jan 13 to Feb 12 | 5.71 | 73.57 |
| Libya | Northern Africa | From Jan 13 to Feb 12 | 26.67 | 97.14 |
| Sudan | Northern Africa | From Jan 13 to Feb 12 | 14.29 | 90.00 |
| Finland | Northern Europe | From Jan 13 to Feb 12 | 9.52 | 75.71 |
| Iceland | Northern Europe | From Jan 13 to Feb 12 | 9.52 | 58.57 |
| Ireland | Northern Europe | From Jan 13 to Feb 12 | 9.52 | 86.67 |
| Norway | Northern Europe | From Jan 13 to Feb 12 | 14.29 | 85.24 |
| United Kingdom | Northern Europe | From Jan 13 to Feb 12 | 14.29 | 80.95 |
| Australia | Oceania | From Jan 13 to Feb 12 | 9.52 | 68.1 |
| New Zealand | Oceania | From Jan 13 to Feb 12 | 18.1 | 97.14 |
| Papua New Guinea | Oceania | From Jan 13 to Feb 12 | 14.29 | 97.14 |
| Argentina | South America | From Jan 13 to Feb 12 | 14.29 | 97.14 |
| Colombia | South America | From Jan 13 to Feb 12 | 9.52 | 92.38 |
| Ecuador | South America | From Jan 13 to Feb 12 | 14.29 | 97.14 |
| Guyana | South America | From Jan 13 to Feb 12 | 2.86 | 92.38 |
| Paraguay | South America | From Jan 13 to Feb 12 | 14.29 | 92.38 |
| Indonesia | Southeast Asia | From Jan 13 to Feb 12 | 2.86 | 75.24 |
| Malaysia | Southeast Asia | From Jan 13 to Feb 12 | 14.29 | 80.00 |
| Philippines | Southeast Asia | From Jan 13 to Feb 12 | 14.29 | 94.29 |
| Vietnam | Southeast Asia | From Jan 13 to Feb 12 | 2.86 | 97.14 |
| South Africa | Southern Africa | From Jan 13 to Feb 12 | 2.86 | 92.38 |
| Bangladesh | Southern Asia | From Jan 13 to Feb 12 | 2.86 | 94.29 |
| India | Southern Asia | From Jan 13 to Feb 12 | 6.43 | 97.14 |
| Pakistan | Southern Asia | From Jan 13 to Feb 12 | 8.57 | 97.14 |
| Sri Lanka | Southern Asia | From Jan 13 to Feb 12 | 2.86 | 97.14 |
| Bosnia and Herzegovina | Southern Europe | From Jan 13 to Feb 12 | 14.29 | 90.71 |
| Croatia | Southern Europe | From Jan 13 to Feb 12 | 9.52 | 97.14 |
| Italy | Southern Europe | From Jan 13 to Feb 12 | 2.86 | 94.29 |
| Portugal | Southern Europe | From Jan 13 to Feb 12 | 14.29 | 94.29 |
| Serbia | Southern Europe | From Jan 13 to Feb 12 | 2.86 | 97.14 |
| Spain | Southern Europe | From Jan 13 to Feb 12 | 14.29 | 88.81 |
| Gambia | Western Africa | From Jan 13 to Feb 12 | 9.52 | 87.62 |
| Ghana | Western Africa | From Jan 13 to Feb 12 | 2.86 | 85.24 |
| Mauritania | Western Africa | From Jan 13 to Feb 12 | 5.71 | 75.71 |
| Nigeria | Western Africa | From Jan 13 to Feb 12 | 11.43 | 82.38 |
| Azerbaijan | Western Asia | From Jan 13 to Feb 12 | 2.86 | 87.62 |
| Bahrain | Western Asia | From Jan 13 to Feb 12 | 9.52 | 87.62 |
| Israel | Western Asia | From Jan 13 to Feb 12 | 4.76 | 92.38 |
| Kuwait | Western Asia | From Jan 13 to Feb 12 | 9.52 | 90.00 |
| Oman | Western Asia | From Jan 13 to Feb 12 | 9.52 | 93.57 |
| Qatar | Western Asia | From Jan 13 to Feb 12 | 2.86 | 90.00 |
| Saudi Arabia | Western Asia | From Jan 13 to Feb 12 | 14.29 | 93.57 |
| Syria | Western Asia | From Jan 13 to Feb 12 | 2.86 | 94.29 |
| Turkey | Western Asia | From Jan 13 to Feb 12 | 2.86 | 85.24 |
| United Arab Emirates | Western Asia | From Jan 13 to Feb 12 | 2.86 | 88.81 |
| Belgium | Western Europe | From Jan 13 to Feb 12 | 9.52 | 79.29 |
| France | Western Europe | From Jan 13 to Feb 12 | 2.86 | 92.38 |
| Germany | Western Europe | From Jan 13 to Feb 12 | 9.52 | 77.62 |
| Dominican Republic | Caribbean | From Feb 13 to Mar 11 | 14.29 | 97.14 |
| Puerto Rico | Caribbean | From Feb 13 to Mar 11 | 14.29 | 94.29 |
| Belize | Central America | From Feb 13 to Mar 11 | 9.52 | 72.14 |
| Honduras | Central America | From Feb 13 to Mar 11 | 14.29 | 97.14 |
| Mexico | Central America | From Feb 13 to Mar 11 | 2.86 | 89.52 |
| Nicaragua | Central America | From Feb 13 to Mar 11 | 2.86 | 20.00 |
| Kyrgyz Republic | Central Asia | From Feb 13 to Mar 11 | 5.71 | 88.81 |
| Malawi | Eastern Africa | From Feb 13 to Mar 11 | 14.29 | 61.43 |
| South Sudan | Eastern Africa | From Feb 13 to Mar 11 | 5.71 | 97.14 |
| Zambia | Eastern Africa | From Feb 13 to Mar 11 | 20.00 | 60.95 |
| Zimbabwe | Eastern Africa | From Feb 13 to Mar 11 | 9.52 | 92.38 |
| Czech Republic | Eastern Europe | From Feb 13 to Mar 11 | 14.29 | 76.43 |
| Hungary | Eastern Europe | From Feb 13 to Mar 11 | 9.52 | 80.48 |
| Moldova | Eastern Europe | From Feb 13 to Mar 11 | 9.52 | 88.81 |
| Ukraine | Eastern Europe | From Feb 13 to Mar 11 | 14.29 | 93.57 |
| Chad | Middle Africa | From Feb 13 to Mar 11 | 20.00 | 97.14 |
| Greenland | North America | From Feb 13 to Mar 11 | 14.29 | 88.81 |
| Algeria | Northern Africa | From Feb 13 to Mar 11 | 9.52 | 87.14 |
| Morocco | Northern Africa | From Feb 13 to Mar 11 | 9.52 | 97.14 |
| Tunisia | Northern Africa | From Feb 13 to Mar 11 | 17.14 | 97.14 |
| Denmark | Northern Europe | From Feb 13 to Mar 11 | 14.29 | 84.05 |
| Sweden | Northern Europe | From Feb 13 to Mar 11 | 14.29 | 58.1 |
| Brazil | South America | From Feb 13 to Mar 11 | 14.29 | 75.71 |
| Peru | South America | From Feb 13 to Mar 11 | 14.29 | 92.38 |
| Venezuela | South America | From Feb 13 to Mar 11 | 14.29 | 82.86 |
| Thailand | Southeast Asia | From Feb 13 to Mar 11 | 20.00 | 84.05 |
| Eswatini | Southern Africa | From Feb 13 to Mar 11 | 9.52 | 80.00 |
| Lesotho | Southern Africa | From Feb 13 to Mar 11 | 5.71 | 92.38 |
| Namibia | Southern Africa | From Feb 13 to Mar 11 | 2.86 | 75.71 |
| Afghanistan | Southern Asia | From Feb 13 to Mar 11 | 23.81 | 75.48 |
| Iran | Southern Asia | From Feb 13 to Mar 11 | 4.76 | 76.19 |
| Albania | Southern Europe | From Feb 13 to Mar 11 | 2.86 | 85.24 |
| Greece | Southern Europe | From Feb 13 to Mar 11 | 14.29 | 85.95 |
| Kosovo | Southern Europe | From Feb 13 to Mar 11 | 9.52 | 93.57 |
| San Marino | Southern Europe | From Feb 13 to Mar 11 | 20.00 | 84.29 |
| Slovenia | Southern Europe | From Feb 13 to Mar 11 | 14.29 | 94.29 |
| Cyprus | Western Asia | From Feb 13 to Mar 11 | 14.29 | 93.57 |
| Iraq | Western Asia | From Feb 13 to Mar 11 | 8.57 | 89.52 |
| Jordan | Western Asia | From Feb 13 to Mar 11 | 14.29 | 97.14 |
| Lebanon | Western Asia | From Feb 13 to Mar 11 | 2.86 | 88.81 |
| Palestine | Western Asia | From Feb 13 to Mar 11 | 9.52 | 97.14 |
| Austria | Western Europe | From Feb 13 to Mar 11 | 14.29 | 84.76 |
| Luxembourg | Western Europe | From Feb 13 to Mar 11 | 14.29 | 80.95 |
| Netherlands | Western Europe | From Feb 13 to Mar 11 | 4.76 | 84.76 |
| Switzerland | Western Europe | From Feb 13 to Mar 11 | 9.52 | 76.43 |
| Aruba | Caribbean | After Mar 11 | 8.57 | 82.86 |
| Dominica | Caribbean | After Mar 11 | 17.14 | 82.86 |
| Kazakhstan | Central Asia | After Mar 11 | 14.29 | 85.24 |
| Djibouti | Eastern Africa | After Mar 11 | 25.71 | 97.14 |
| Ethiopia | Eastern Africa | After Mar 11 | 45.71 | 78.33 |
| Madagascar | Eastern Africa | After Mar 11 | 8.57 | 97.14 |
| Mauritius | Eastern Africa | After Mar 11 | 8.57 | 93.57 |
| Mozambique | Eastern Africa | After Mar 11 | 9.52 | 55.71 |
| Cameroon | Middle Africa | After Mar 11 | 2.86 | 70.48 |
| Democratic Republic of Congo | Middle Africa | After Mar 11 | 2.86 | 85.24 |
| Egypt | Northern Africa | After Mar 11 | 10.71 | 93.57 |
| Estonia | Northern Europe | After Mar 11 | 45.71 | 80.00 |
| Guam | Oceania | After Mar 11 | 52.14 | 72.86 |
| Bolivia | South America | After Mar 11 | 28.57 | 97.14 |
| Chile | South America | After Mar 11 | 14.29 | 72.86 |
| Uruguay | South America | After Mar 11 | 29.52 | 85.24 |
| Laos | Southeast Asia | After Mar 11 | 16.67 | 97.14 |
| Andorra | Southern Europe | After Mar 11 | 16.67 | 77.62 |
| Burkina Faso | Western Africa | After Mar 11 | 17.14 | 93.57 |
| Cape Verde | Western Africa | After Mar 11 | 14.29 | 88.81 |
| Mali | Western Africa | After Mar 11 | 51.43 | 80.00 |
| Niger | Western Africa | After Mar 11 | 14.29 | 63.81 |
| Sierra Leone | Western Africa | After Mar 11 | 14.29 | 94.29 |
|  | | | | |

| **Additional Table 3. The relationship between time to a high Stringency Index (SI) level and time to a peak daily incidence, when a start of response was redefined as SI>10^*^** | | | |
| --- | --- | --- | --- |
|  | β | Standard error | *p*-value |
| (A) From the start of response (i.e., SI>0) |  |  |  |
| Model 1: Unadjusted | 0.81 | 0.06 | <.0001 |
| Model 2: Model 1+ further adjusted geographic regions, SI level and initiated stage | 0.65 | 0.09 | <.0001 |
| (B) From the first reported case |  |  |  |
| Model 1: Unadjusted | 0.64 | 0.06 | <.0001 |
| Model 2: Model 1+ further adjusted geographic regions, SI level and initiated stage | 0.62 | 0.07 | <.0001 |
| ^*^Multivariable linear regression models were used to analyze the association. | | | |


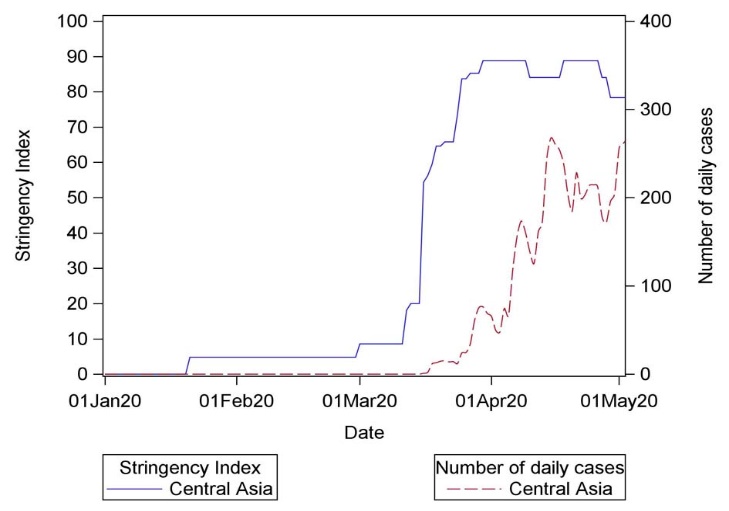

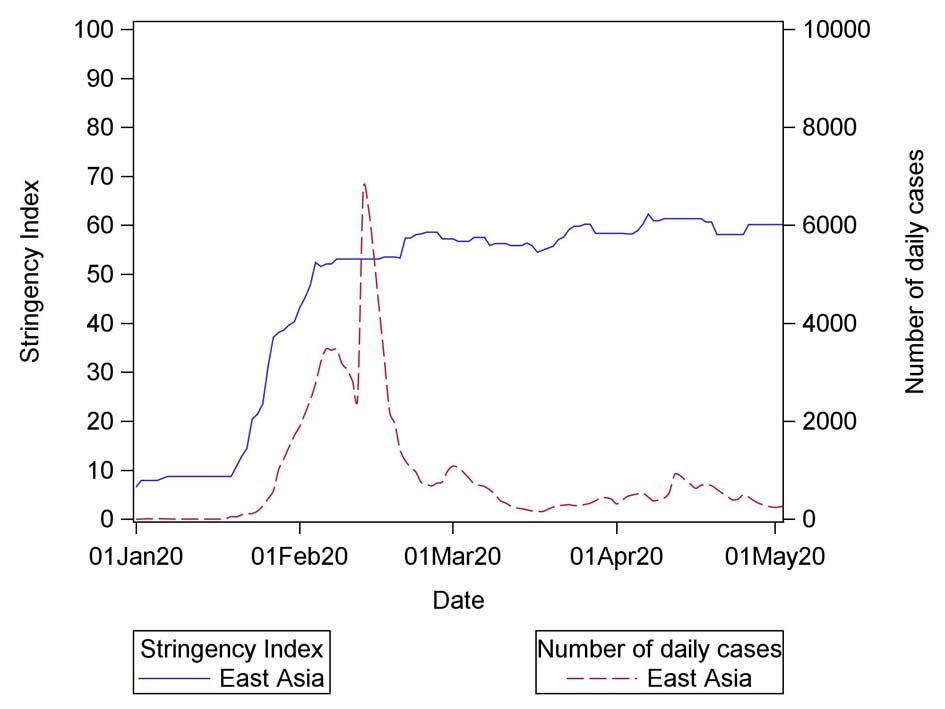

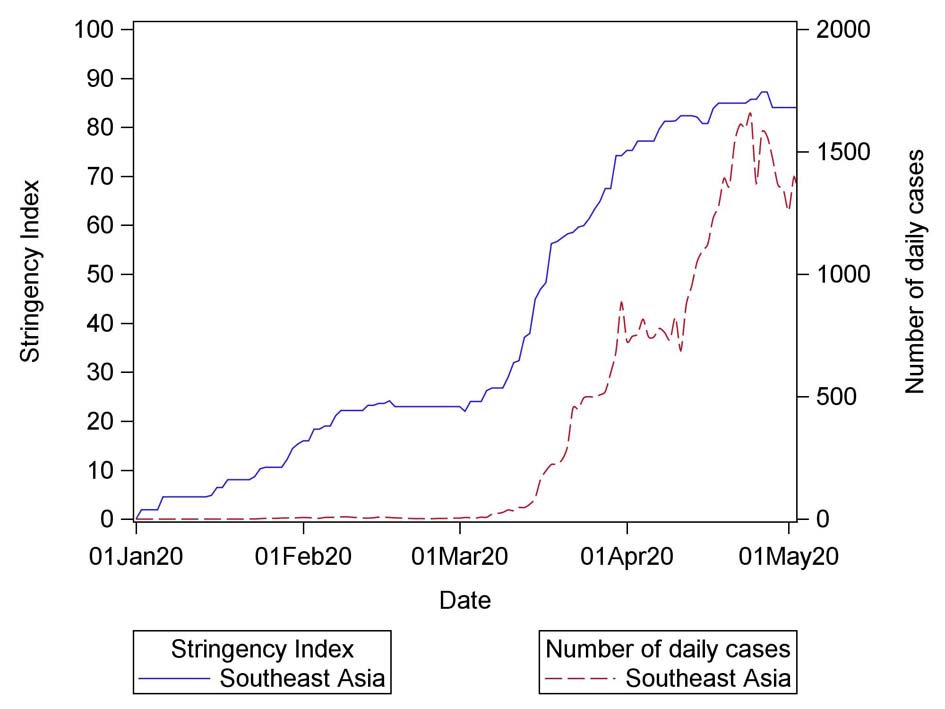

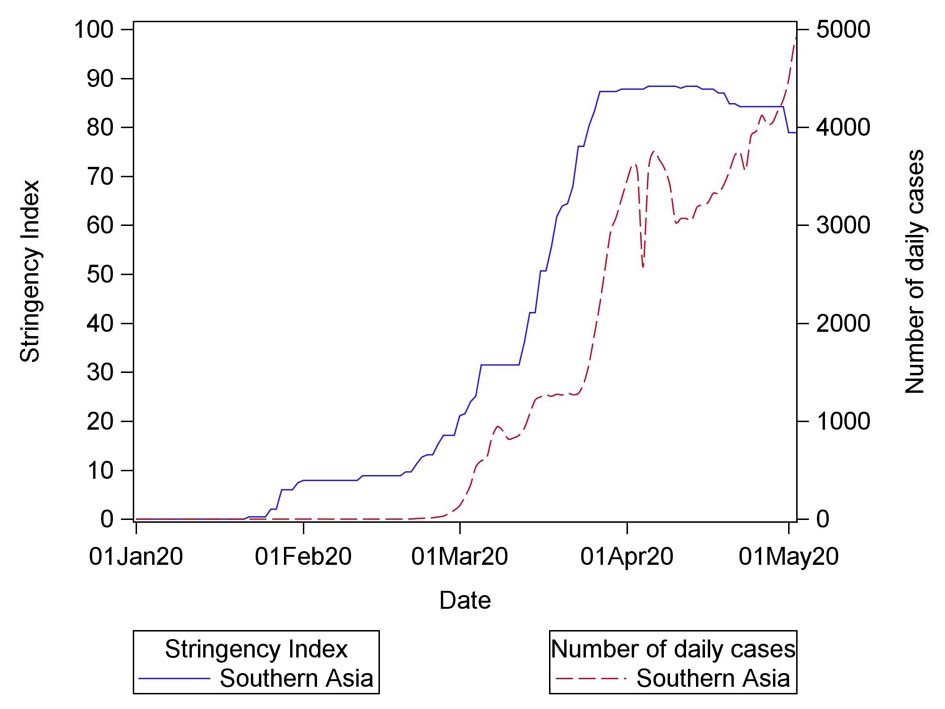

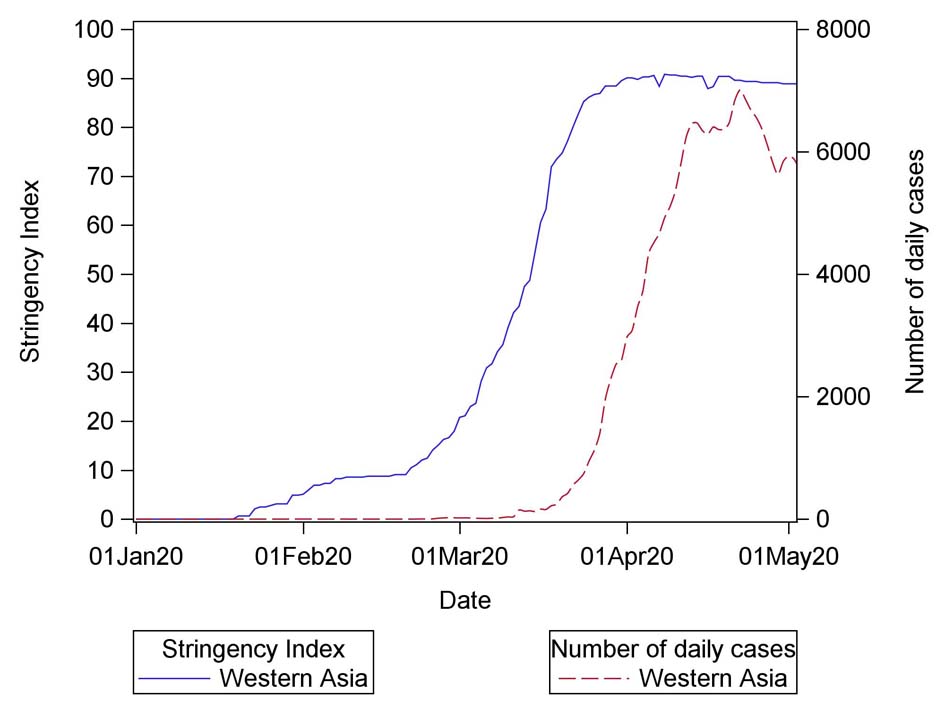


**Additional Fig. 1-A** Trajectories of Stringency Index and number of daily new cases in Asia (An exponentially weighted moving average method with parameter 0.3 was used to smooth time series of Stringency Index and number of daily new cases, and a base-10 log scale was used for the Y axis of number of daily new cases. Similar methods were used for Figure 6-B, C and D)


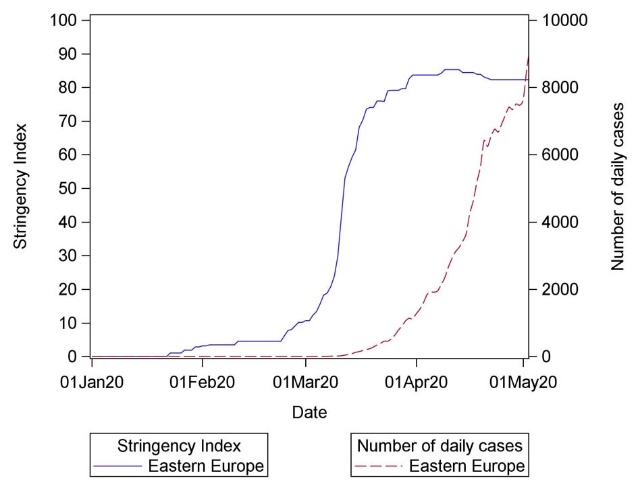

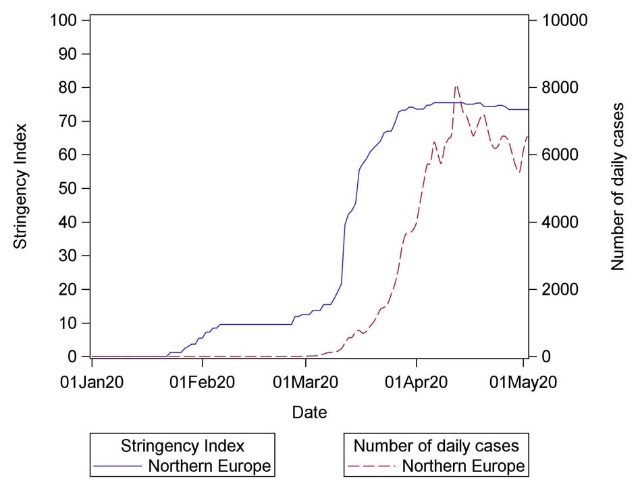

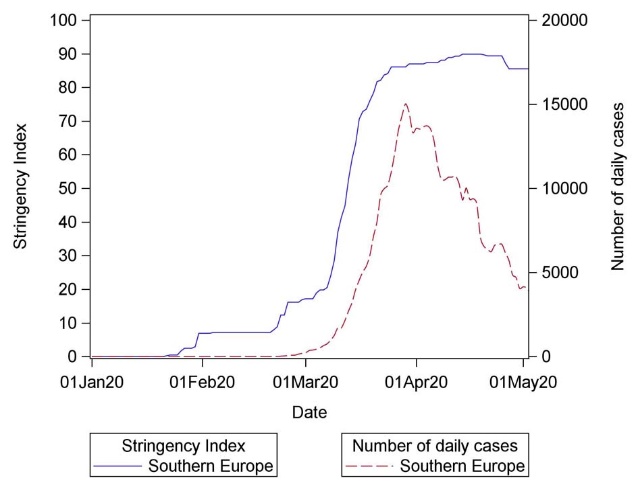

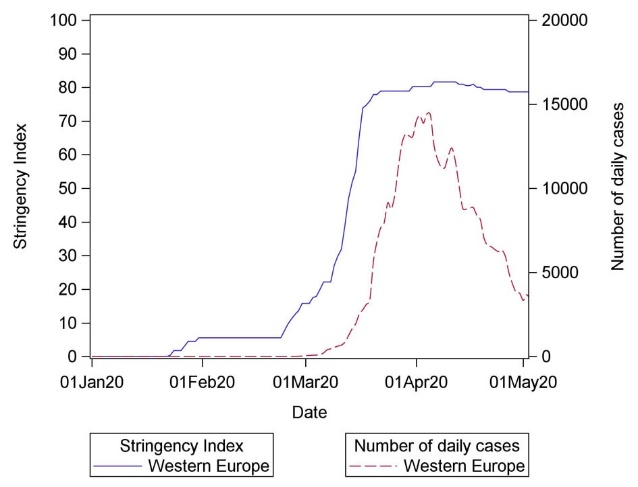


**Additional Fig. 1-B** Trajectories of Stringency Index and number of daily new cases in Europe


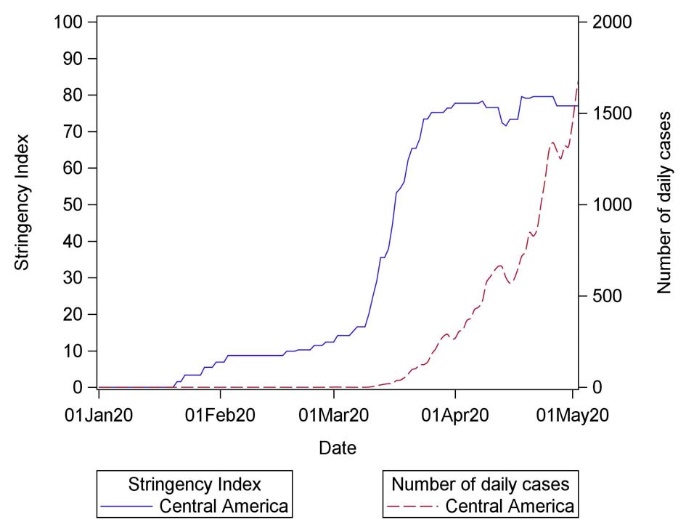

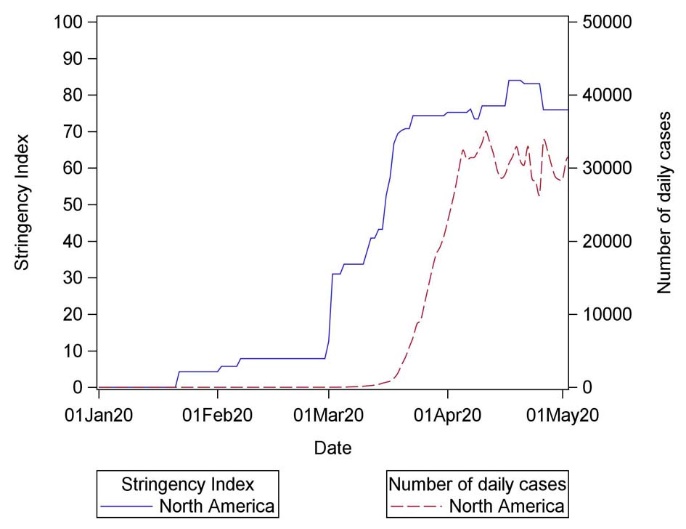

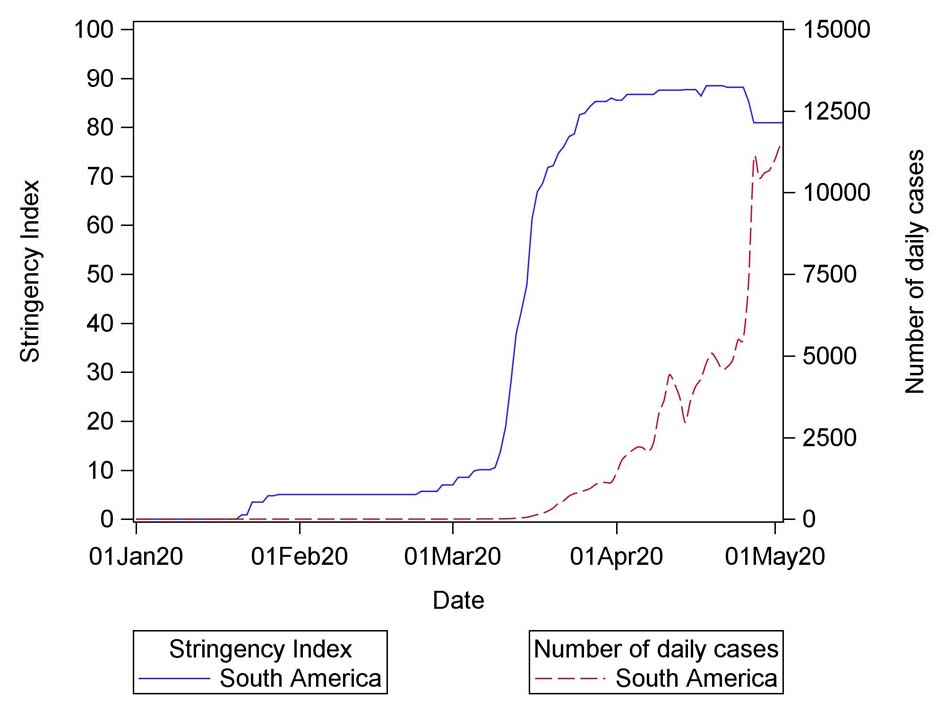

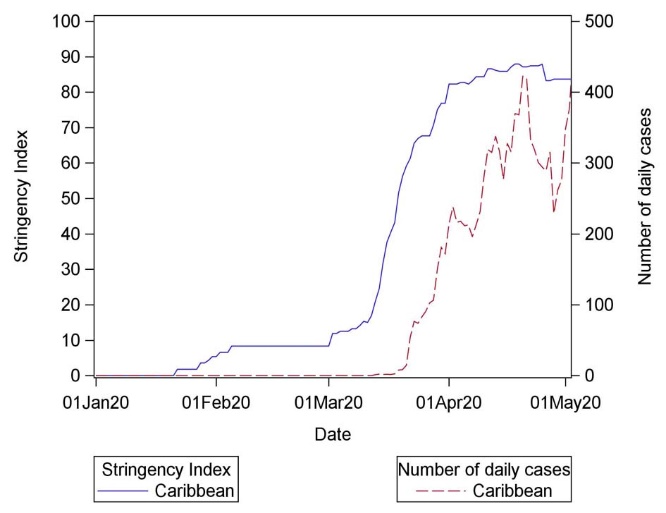

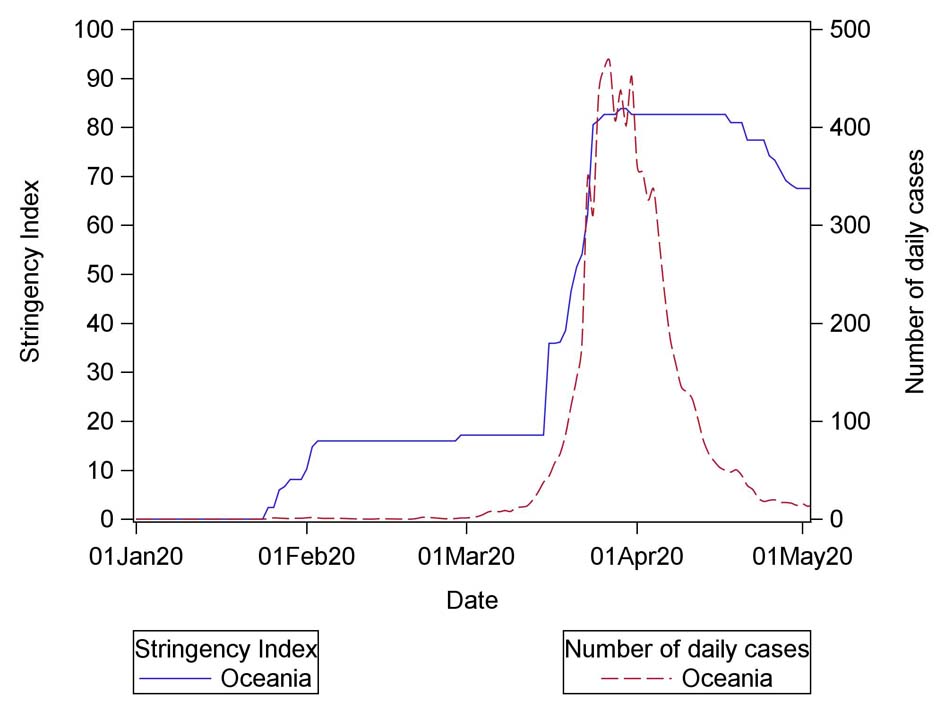


**Additional Fig. 1-C** Trajectories of Stringency Index and number of daily new cases in America, Oceania and Caribbean


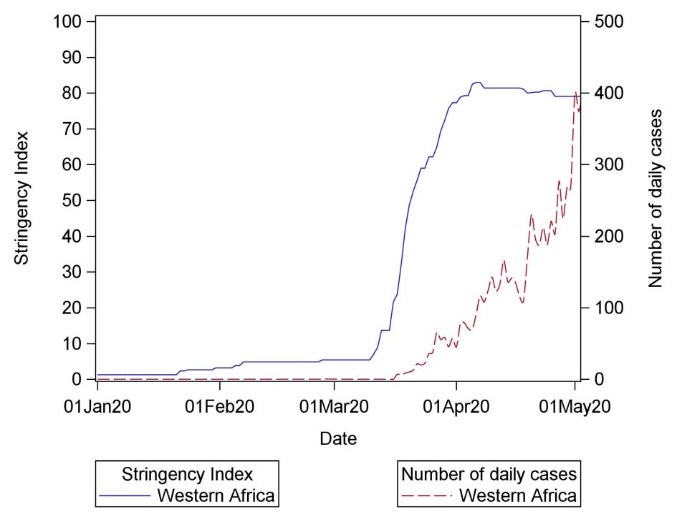

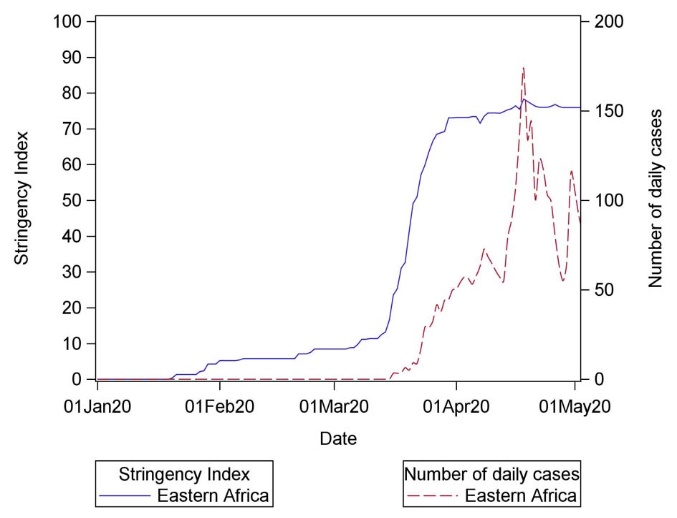

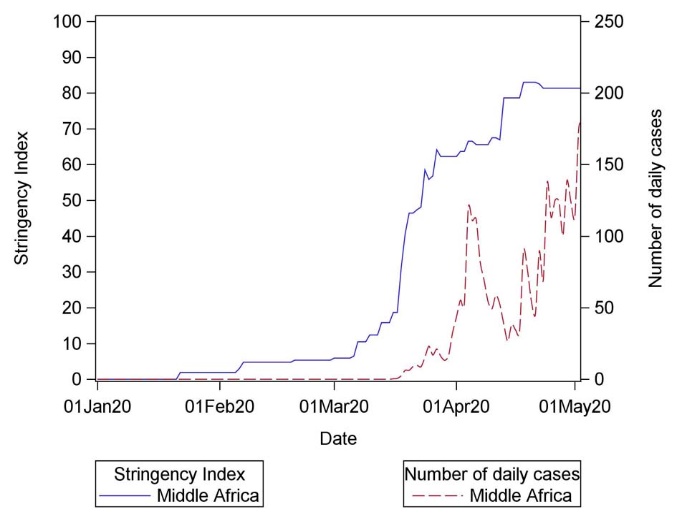

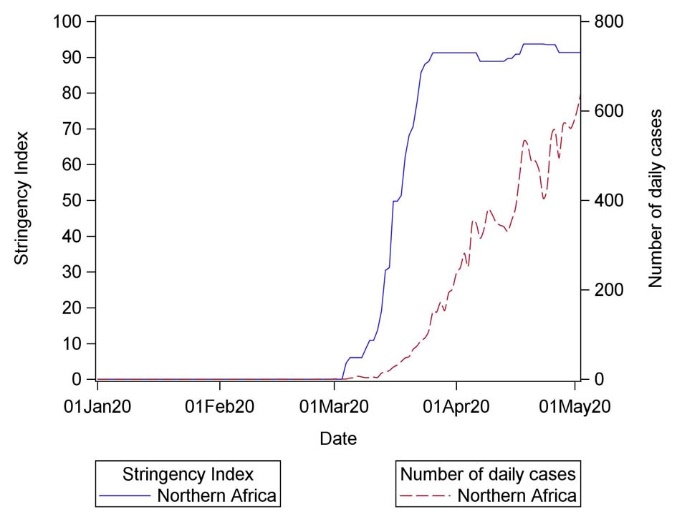

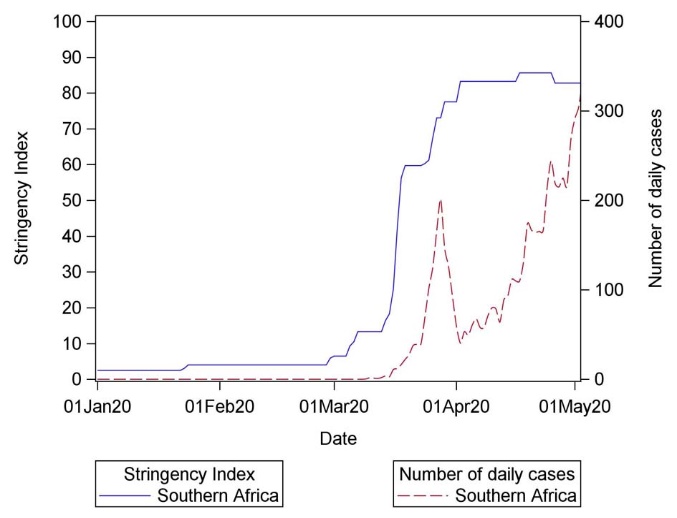


**Additional Fig. 1-D** Trajectories of Stringency Index and number of daily new cases in Africa
